# 871. Evolution of Multi-specialty Collaboration in Burn Surgery Research Across Four Decades

**DOI:** 10.1093/jbcr/irag033.348

**Published:** 2026-04-06

**Authors:** Francesco M Egro, Kian Daneshi, Sarah M Tepe, Mare G Kaulakis, Christopher J Fedor

**Affiliations:** University of Pittsburgh Medical Center, Pittsburgh, PA; School of Population Health and Medicine, University of Sheffield, London, England; University of Pittsburgh Medical Center, Pittsburgh, PA; University of Pittsburgh Medical Center, Mercy Burn Center, Pittsburgh, PA; University of Pittsburgh Medical Center, Department of Plastic Surgery, Pittsburgh, PA

## Abstract

**Introduction:**

Burns care requires extensive multi-disciplinary expertise spanning surgical, medical and allied health specialties. While collaboration is fundamental to clinical practice, systematic analysis of multi-specialty academic partnerships in burns research has been limited. Understanding the specific patterns of interdisciplinary involvement provides crucial insights into how collaborative clinical care models translate into academic medicine.

**Methods:**

We analyzed 18 275 articles from 11 burns journals(1982-2025) using automated PubMed data mining to identify collaborative publications. Specialty involvement was determined through systematic affiliation analysis across all papers. Multi-specialty combinations were categorized to assess percentage involvement of multiple specialties. Statistical analysis included chi-square tests for specialty involvement patterns and linear regression for temporal trends.

**Results:**

Among 14 675 analyzable papers, multi-specialty involvement affected 15.6% of all burns literature, demonstrating substantial interdisciplinary collaboration across the field. Plastic surgery appeared in 28.6% of all papers, general surgery in 19.4%, with combined plastic-general surgery collaboration comprising 5.5% of the entire corpus (805 papers). Plastic surgery-general surgery papers were significantly more likely to involve additional specialties than single-department publications (OR = 1.64, χ^2^ = 33.543, *p*<.001). The most common multi-specialty combination was plastic + general surgery (4.05% of all papers). Individual specialty involvement revealed: pediatrics (9.3% of all papers), critical care (5.2%), physical therapy (4.5%), anesthesia (3.7%). Temporal analysis demonstrated dramatic growth in multi-specialty collaboration: from 0.9% of papers (1980s) to 27.8% (2020s), representing a 30-fold increase in interdisciplinary partnerships over four decades.

**Conclusions:**

This analysis reveals that collaboration in burns surgery extends far beyond clinical practice into academic medicine, with systematic patterns of multi-specialty involvement reflecting the complexity of burn care. The dramatic 30-fold increase in multi-specialty publications demonstrates the field's evolution toward integrated care models. Nearly a fifth of all burns literature now involves multiple specialties, with plastic surgery-general surgery partnerships serving as collaboration catalysts that significantly increase likelihood of additional specialty involvement. These findings establish burns surgery as a model for multi-specialty academic collaboration in reflecting real-life clinical practice.

**Applicability of Research to Practice:**

This research highlights the importance of multi-specialty collaboration in burn care. There is a push for collaboration in medicine among specialties. Allowing for strong relationships between specialties will ultimately lead to better patient care.

**Funding for the study:**

N/A.

